# Supplementary material for: Transferring Knowledge on Motor Development to Socially Vulnerable Parents of Infants: The Practice of Health Visitors
Source: Int J Environ Res Public Health. 2021 Nov 25;18(23):12425. doi: 10.3390/ijerph182312425 (PMC8657119; doi:10.3390/ijerph182312425)
Supplement: Supplementary file 1 [file ijerph-18-12425-s001.zip › ijerph-1405643-supplementary.pdf]

Supplementary Materials:

The *´Integrated Implementation model´*. The focus areas addressed in the article are highlighted in red.

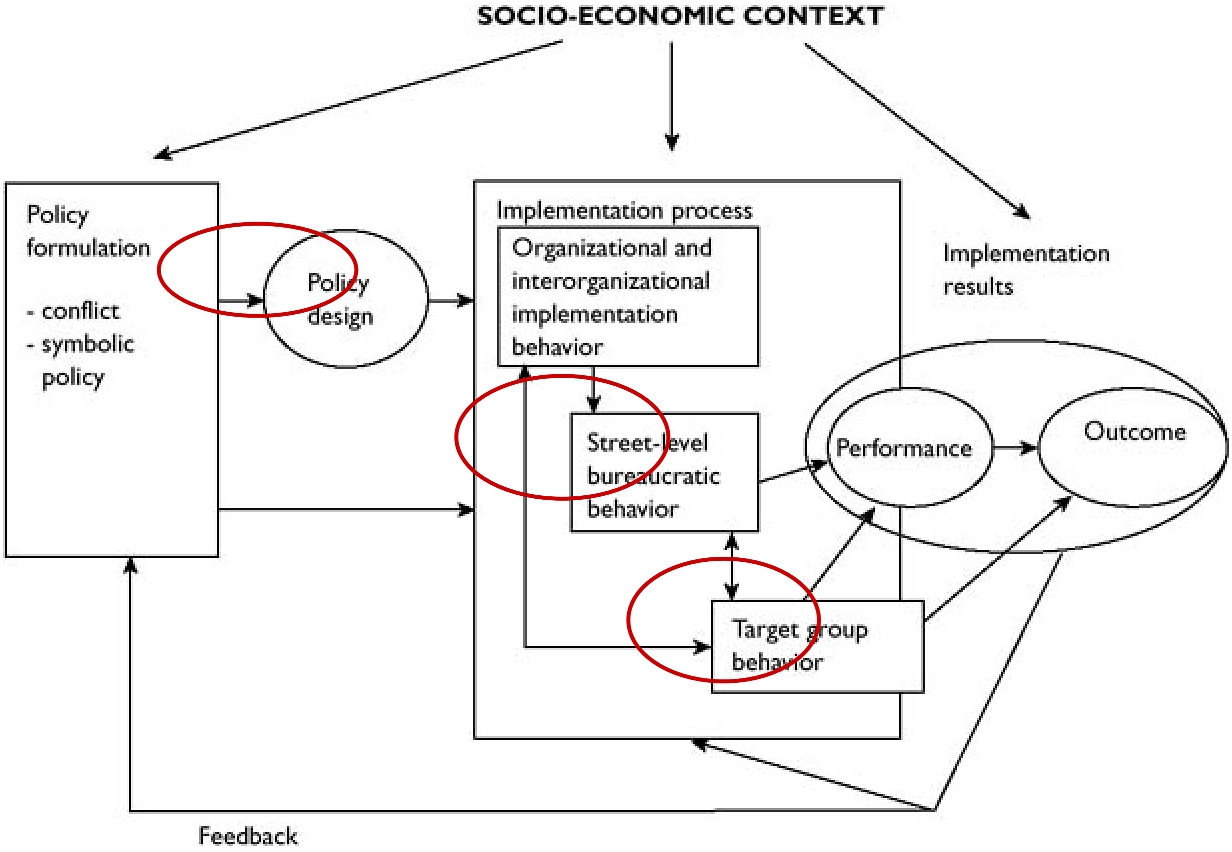

Figure S1. The Integrated Implentation model of Winter and Nielsen. Handbook of Public Administration (2003). See Chapter 5: Innovations and Global Trends in Human Resource Management Practices, doi:10.4135/9781848608214.
